# School-based assessment of amblyopia and strabismus among multiethnic children in rural China

**DOI:** 10.1038/s41598-017-13926-8

**Published:** 2017-10-17

**Authors:** Chen-Wei Pan, Xuejuan Chen, Hui Zhu, Zhujun Fu, Hua Zhong, Jun Li, Dan Huang, Hu Liu

**Affiliations:** 1 School of Public Health, Medical College of Soochow University, Suzhou, China; 2 Jiangsu Key Laboratory of Preventive and Translational Medicine for Geriatric Diseases, Soochow University, Suzhou, China; 3 Department of Ophthalmology, the First Affiliated Hospital of Nanjing Medical University, Nanjing, China; 4 Department of Ophthalmology, Nanjing Children’s Hospital, Nanjing, China; 5 Department of Ophthalmology, First Affiliated Hospital of Kunming Medical University, Kunming, China; 6 Department of Ophthalmology, the Second People’s Hospital of Yunnan Province, Kunming, China

## Abstract

We aimed to determine the prevalence and possible ethnic variations in strabismus and amblyopia among multiethnic school-aged children in rural China. A total of 9,263 children (4,347 Han, 3,352 Yi, 799 Dai and 765 Bai) aged 6 to 14 years were analyzed. Comprehensive eye examinations including monocular distance visual acuity, anterior segment examination, autorefraction, cover testing and ocular motility were conducted. Manifested strabismus was detected in 3.53% of the overall population. The prevalence of strabismus was 3.29% in Han, 4.12% in Yi, 2.25% in Dai, and 3.66% in Bai ethnic groups with marginally inter-ethnic differences (*P* = 0.046). There was an increasing trend in the prevalence of strabismus with increasing age (P < 0.001). Amblyopia affected 132 children (1.43%) overall, with no statistical differences in gender and age. The prevalence of amblyopia was highest in Dai ethnic group (2.00%) and lowest in ethnic Yi ethnic group (1.04%) with no significant difference being detected (*P* = 0.062). Refractive error and strabismus were the two major factors associated with amblyopia. No significant ethnic differences in strabismus and amblyopia among Chinese ethnic minorities were observed. Refractive error and strabismus were the major causes for amblyopia in rural Chinese children.

## Introduction

Amblyopia and strabismus are two major childhood-onset ocular disorders exerting persistent negative effects on visual and binocular function development. Strabismus manifests as a condition in which the eyes are improperly aligned. The condition is identified as a common cause of amblyopia in children, presenting significant psychosocial and psychosocial consequences followed by adverse effects on binocularity, stereopsis, and depth of perception^1,2^. Amblyopia refers to unilateral or bilateral reduction in best corrected visual acuity (BCVA) not directly attributable to structural abnormality of the eye or posterior visual pathways. Individuals with childhood-onset unilateral amblyopia have a greater lifetime risk of eventual bilateral visual impairment and age-related macular degeneration^3^. Nevertheless, amblyopia is potentially reversible, if properly treated. Earlier reports have indicated that the treatment of amblyopia is ineffective after 8 years old; yet, recent amblyopia treatment studies have shown that visual acuity can be improved in older children^4^.

Epidemiologic studies on strabismus and amblyopia in preschool and school-aged populations have been widely carried out in recent decades including the Multi-Ethnic Pediatric Eye Disease Study (MEPEDS)^5,6^; the Baltimore Pediatric Eye Disease Study^7^; the Strabismus, Amblyopia, and Refractive Error in Singaporean Children Study^8^; and the Vision In Preschoolers Study^9,10^. In these studies, racial/ethnic differences were observed including higher exotropia versus esotropia ratios as well as lower strabismus and amblyopia prevalence rates in the Asians compared with other ethnic groups.

China is one of the largest developing countries throughout the world with a multiethnic population including Han ethnicity and other 55 ethnic minorities. Han ethnicity is the major ethnic group which accounts for about 90% of the entire national population. There are few data addressing the epidemiology of strabismus and amblyopia among Chinese children of ethnic minorities. Yunnan Province is located on the border of southwestern China and has the largest number of ethnic minorities among all provinces in China, which provides us with a unique opportunity to look into the possible ethnic differences among various Chinese ethnic minorities. In this study, we compared the prevalence and subtypes of strabismus and amblyopia in school-based samples of different ethnic groups including Han, Yi, Dai and Bai Chinese children and adolescents aged 6 to 14 years in rural communities in Yunnan Province.

## Methods

### Study Population

The Yunnan Minority Children Eye Study was a series of school-based studies conducted from 2014 to 2015 in Yangbi and Mangshi in Yunnan Province. The current study was conducted to compare the variations in the prevalence of strabismus and amblyopia among different ethnic groups aged 6 to 14 years in Yangbi and Mangshi located within Yunnan. All 13,738 students from 55 schools located in the study sites were invited to participate in the study, among whom, 10,244 had completed eye examinations and provided parents’ consent, with questionnaire data provided (74.6% response rate). There were no significant differences in terms of age (P = 0.23) and gender (P = 0.55) between responders and non-responders. The study sample included 4,347 Han (42.4%), 3352 Yi (32.7%), 799 Dai (7.8%), 765 Bai (7.5%) and 981 other ethnic minorities (9.6%), with each ethnic group having similar proportions of boys and girls (Table 1). Participants of different ethnicities resided together in the same communities. In the current analysis of the prevalence across ethnic groups, we only included the four major ethnic groups including Han, Yi, Dai and Bai. Some ethnic group such as Jingpo, Hui, Achang, Lisu and other unknown minorities was excluded from the analysis because of the complexity of ethnic composition and inadequate number in each ethnic group. Finally, 9,263 study participants were included in the current analysis. The subjects aged from 6 to 14 years, with a mean age of 9.55 ± 1.93 years, while sex was distributed equally in each of the four ethnic group (Table 1). Ethnicity of the offspring was identified according to the father’s ethnicity.

**Table 1:** Age, gender and ethnic frequency distributions of the elementary school children in the study.

Classification	Han	Yi	Dai	Bai	Total
**Total**	4347	3352	799	765	9263
**mean age (years)**	9.23 ± 1.85	10.09 ± 1.95	8.73 ± 1.63	9.85 ± 1.86	9.55 ± 1.93
**Gender**					
Boys	2261	1692	406	374	4733
Girls	2086	1660	393	391	4530
**Age (years)**					
6	231	30	62	6	329
7	713	453	148	104	1418
8	725	386	163	101	1375
9	752	357	163	117	1389
10	783	538	139	125	1585
11	519	602	81	129	1331
12	496	679	41	138	1354
13	116	272	2	42	432
14	12	35	0	3	50

Written informed consent was obtained from at least one parent after the nature of the study was explained and verbal consent was obtained from each child before examination. All procedures were reviewed and approved by the Kunming Medical University institutional ethical review board in accordance with the Declaration of Helsinki. The study methods were carried out in accordance with the approved guidelines.

### Questionnaire and Eye Examinations

Questionnaires were distributed to all enrolled children who were required to fill out the questionnaire under their parents’ supervision. Basic demographic information as well as medical history of amblyopia, strabismus, and any prior ophthalmic treatment was included in the questionnaire.

A comprehensive ophthalmologic examination including monocular distance visual acuity (VA), anterior segment, ocular alignment, and non-cycloplegic autorefraction was performed by two optometrists and two ophthalmologists who were trained and certified using standardized study protocols, as previously described in MEPEDS^11^. Post-cycloplegic refraction and fundus examination were also performed. The same study ophthalmologists (HZ and ZF) performed the ophthalmologic examinations in all schools.

Ocular alignment was assessed using the Hirschberg light reflex test, the unilateral cover (cover–uncover) test, and the alternate cover test. The cover test was conducted at both distances, namely, distant (6 m) and near (33 cm), by using fixation targets. Visual acuity with and without optical correction was examined if glasses were worn. Ocular movements (versions and ductions) were assessed in nine diagnostic positions of gaze with the head in a stationary position. If strabismus was suspected, a prism cover test was performed to detect the magnitude of the deviation.

Distance VA was measured with a logarithm of the minimum angle of resolution (LogMAR) visual acuity chart (Precision Vision, La Salle, IL, USA) at a distance of 4 m, with or without spectacles. For children with initial VA less than 20/25 in either eye (or 2-line interocular difference), cycloplegic refraction was performed. Subjective refraction was then performed to obtain BCVA. If glasses were worn, testing was performed with and without correction.

The refraction status of all participants was measured using an auto refractometer (NIDEK ARK-510A, NIDEK Corporation, Japan) under non-cycloplegic conditions. The average of three consecutive readings of sphero-cylindrical autorefraction was determined. Spherical refraction (SE) was calculated as the sum of the spherical and half of the cylindrical power. Any child with abnormal ocular alignment, ocular movement, or distance VA was further evaluated by post-cycloplegic refraction. For cycloplegic refraction, one drop of topical 1.0% cyclopentolate was administered to each eye twice with a 5-minute interval. Fifteen minutes later, a third drop was administered if the pupil size was less than 6 mm or if the pupillary light reflex was still present.

### Definitions

Strabismus was defined as any tropia presented at distant or near fixations with or without spectacles. Constant heterotropia was considered as constant tropia at both near and distant fixation; otherwise, it was considered intermittent. Strabismus was defined as constant or intermittent heterotropia of any magnitude at distant or near fixation. Strabismus was classified by the primary direction (esotropia, exotropia, vertical) of the tropia. Microstrabimus is defined as a deviation of fewer than 10 prism diopters, in addition to binocular vision.

Unilateral amblyopia was defined if the participant had a BCVA ≤ 20/32 in the worse eye and a 2-line interocular difference or more in BCVA, without any underlying structural abnormality of the visual pathway and presenting at least one of the following risk factors in the affected eye: past or present strabismus; previous strabismus surgery; anisometropia consistent with the worse eye such as spherical equivalent (SE) difference of ≥3.00 diopters (D) in myopia, ≥1.00 D in hyperopia, or ≥1.50 D in astigmatism; and evidence of past or present visual axis obstruction (e.g., congenital cataract, intraocular lens, aphakia, corneal opacity, ptosis, or eyelid hemangioma). Bilateral amblyopia was defined as a condition of bilateral subnormal BCVA < 20/40, presenting amblyopia factors including bilateral high ametropia (myopia ≥ 6.00 D, hyperopia ≥ 4.00 D, or astigmatism ≥ 2.50 D) and evidence of past or present visual axis obstruction (see above). Children who met the unilateral and bilateral amblyopia criteria were classified as unilateral amblyopia, similar to that used in MEPDES^5,6^.

### Statistical Analysis

Statistical analyses were performed using a commercially available statistical software package (SPSS for Windows, version 19.0, IBM–SPSS, China). The prevalence rates of strabismus and amblyopia were calculated by age and gender for each of the four ethnic groups. The chi-square test incorporating sampling weights with a 0.05 significance level was used to compare the prevalence rates for the four ethnic groups. Odds ratios (ORs) and 95% confidence intervals (CIs) were also presented.

## Results

Table 2 shows the prevalence of strabismus by age and gender. On the basis of the comprehensive eye examinations of 9,263 subjects, manifest strabismus was detected in 327 children [3.53%, 95% Confidence Interval (CI), 3.15–3.91%], including 143 Han, 138 Yi, and 18 Dai and 28 Bai children, with corresponding prevalence rates of 3.29% (95% CI, 2.76–3.82%), 4.12% (95% CI, 3.44–4.79%), 2.25% (95% CI, 1.22–3.28%), and 3.66% (95% CI, 2.33–4.99%), respectively. Marginally significant ethnic differences in the prevalence of strabismus were indicated, with a *P* value of 0.046 (Table 2). The overall sample was divided into four three groups by age (6 to 8 years, 9 to 11 years, and 12 to 14 years). Higher prevalence of strabismus was found in older age groups, ranging from 2.47% to 4.96% (*P* < 0.001). Gender was not associated with the prevalence of strabismus (*P* = 0.49).

**Table 2:** Prevalence of strabismus and amblyopia in the elementary school children in the study.

Classification	Strabismus				Amblyopia			
	With	Without	Prevalence, 95% CI (%)	P value	With	Without	Prevalence, 95% CI (%)	P value
**Overall**	327	8936	3.53 (3.15–3.91)		132	9131	1.43 (1.18–1.67)	
**Gender**				0.493				0.192
Boys	161	4572	3.40 (2.89–3.92)		60	4673	1.27 (0.95–1.59)	
Girls	166	4364	3.66 (3.12–4.21)		72	4458	1.59 (1.23–1.95)	
**Age (years)**				0.000				0.704
6 to 8	77	3045	2.47 (1.92–3.01)		49	3073	1.57 (1.13–2.01)	
9 to 11	159	4146	3.69 (3.13–4.26)		58	4247	1.35 (1.00–1.69)	
12 to 14	91	1745	4.96 (3.96–5.95)		25	1811	1.36 (0.83–1.89)	
**Ethnicity**				0.046				0.062
Han	143	4204	3.29 (2.76–3.82)		72	4275	1.66 (1.28–2.04)	
Yi	138	3214	4.12 (3.44–4.79)		35	3317	1.04 (0.70–1.39)	
Dai	18	781	2.25 (1.22–3.28)		16	783	2.00 (1.03–2.97)	
Bai	28	737	3.66 (2.33–4.99)		9	756	1.18 (0.41–1.94)	

Table 3 demonstrates the distribution of strabismus subtypes. Among the 327 children with strabismus, 3 reported a history of strabismus surgery, 2 presented with normal ocular position, and 1 had residual exotropia. Among strabismic children in each ethnic group, the number of children with exotropia remarkably exceeded the number of those with esotropia, with exotropia versus esotropia ratios of 10.5:1 (115:11) in ethnic Han, 10.3:1 (113:11) in ethnic Yi, 7.5:1 (15:2) in ethnic Dai and 7:1 (21:4) in ethnic Bai.

**Table 3:** Subtypes and magnitude of strabismus in 5 ethnic groups.

Classification	Han	Yi	Dai	Bai	*p* value
Any strabismus, N(%)(95% CI)	143 (3.29) (2.76–3.82)	138 (4.12) (3.44–4.79)	18 (2.25) (1.22–3.28)	28 (3.66) (2.33–4.99)	0.046
Exotropia	115 (2.65) (2.17–3.12)	113 (3.37) (2.76–3.98)	15 (1.88) (0.94–2.82)	21 (2.75) (1.59–3.90)	0.082
Esotropia	11 (0.25) (0.10–0.40)	11 (0.33) (0.13–0.52)	2 (0.25) (0.00–0.60)	4 (0.52) (0.01–1.03)	0.630
Rate of Exotropia: Esotropia	10.5: 1	10.3: 1	7.5: 1	7.0: 1	
**Subtypes**					
Intermittent exotropia	98	103	10	19	
Constant exotropia	17	10	5	2	
Intermittent esotropia	6	2	2	0	
Constant esotropia	5	9	0	4	
Pure vertical strabismus	17	12	1	3	
Type I Duane syndrome	0	2	0	0	
**Magnitude at distance (horizontal SPCT)**					
1 to 9 PD	8	10	2	2	
10 to 30 PD	79	95	7	19	
>30 PD	22	19	6	4	
Unable to measure	17	0	2	0	
**Magnitude at near (horizontal SPCT)**					
1 to 9 PD	49	50	6	10	
10 to 30 PD	44	55	6	9	
>30 PD	16	19	3	5	
Unable to measure	17	0	2	1	

Among the 9,263 children, 72 Han (1.66%), 35 Yi (1.04%), 16 Dai (2.00%), and 9 (1.18%) Bai children were found to be affected by amblyopia (Table 2). The overall prevalence of amblyopia in this study was 1.43% (95% CI, 1.18–1.67%). No differences were observed in the prevalence of amblyopia in gender (*P* = 0.192) or age (*P* = 0.704). Bilateral amblyopia was present in 8 (0.09%) children, whereas unilateral amblyopia was observed in 124 (1.34%) children.

The distribution of the types of amblyopia by ethnic groups is presented in Table 4. Refractive error was identified as the most frequent cause of unilateral amblyopia and was present in 103 cases (83.06%). Combined strabismic/anisometropic ranked the second and was observed in 16 cases (12.90%), followed by strabismus in 5 cases (4.03%). Therefore, 119 cases of all amblyopia cases were attributable to abnormal refractive error. Only 21 cases were attributable, even in part, to strabismus. On the basis of questionnaire information, 5 children were previously diagnosed with and treated for amblyopia, 2 of whom remained amblyopic upon our examination.

**Table 4:** Types of Amblyopia.

Classification	Han	Yi	Dai	Bai	Total
**Unilateral**	68	32	15	9	124
Anisometropic	58	25	13	7	103
Strabismic	2	2	0	1	5
Combined strabismic/anisometropic	8	5	2	1	16
**Bilateral**	4	3	1	0	8
Refractive	4	3	1	0	8

## Discussion

This study provides new school-based data on the prevalence and ethnic variations in strabismus and amblyopia in multiethnic school-aged children in rural China. Overall, the ethnic differences in strabismus and amblyopia in Chinese ethnic minorities was not pronounced. Intermittent exotropia was the predominant form of strabismus in each ethnic group, whereas the extropia versus estropia ratios were high.

Our study filled the gap of knowledge on the epidemiology of major pediatric eye disorders in rural China. A detailed comparison of prevalence estimates between our study and previous ones is summarized in Table 5. The overall prevalence of strabismus in our study was higher compared with that in Turkey (2.4%; 6 to 14 years)^12^, Japan (1.3%; 6 to 12 years)^13^ and the RESC studies (5 to 15 years) which reported the prevalence of strabismus of 1.9% in southern China^14^, 1.3% in South Africa^15^, 1.9% in India^16^, 0.53% in New Delhi^17^, 2.3% in Chile^18^, and 2.1% in Nepal^19^. When age was restricted to the same range, the prevalence of strabismus in children aged 7 years was similar with that in the United Kingdom (2.68% versus 2.3%)^20^. Meanwhile, the prevalence in children aged 12 years to 13 years (4.93%) was similar with that in the Anyang Childhood Eye Study (5.0%)^21^ but higher than that reported in the Mexican (2.3%)^22^ and Sydney (2.8%)^23^. Considering the variations in methodological issues such as different age ranges of the study participants and definitions of diseases, whether Chinese children had a higher prevalence of strabismus remains unclear.

**Table 5:** Prevalence of Amblyopia and Strabismus in Various Studies.

Studies	Age range (years)	Area	Racial/Ethnic Population	Strabismus prevalence	Exotropia: Esotropia rate	Amblyopia prevalence	Definition of Amblyopia
YMCES	6 to 14	China	Han	3.29%	10.5:1	1.66%	Unilateral: BCVA ≤ 20/32 in the worse eye or 2-line interocular difference in BCVA presenting risk factors; Bilateral: bilateral BCVA < 20/40, presenting amblyopia factors.
			Yi	4.12%	10.3:1	1.04%	
			Dai	2.25%	7.5:1	2.00%	
			Bai	3.66%	7.0:1	1.18%	
			**Multiethnic Chinese**	**3.53%**	**9.4:1**	**1.43%**	
RESC	5 to 15	Shunyi, China	Northern Chinese	2.8%	—	0.92%	BCVA ≤ 20/40 in at least 1 eye associated with amblyopia factors
		Guangzhou, China	Southern Chinese	1.9% at near; 3.0% at distance	almost 80% were extropia	0.87%	
		South Africa	African	1.3% at near; 1.1% at distance	2.8:1 at near; 2.9:1 at distance	0.28%	
		India	Indian	1.9% at near; 1.8% at distance	almost 72% were extropia	0.78%	
		New Delhi, India	Indian	0.53%	1:1.6	0.81%	
		Chile	Hispanic	2.3%	—	2.3%	
		Nepal	Nepali	2.1%	almost all were extropia	1.43%	
Ihsan Caca *et al*.	6 to 14 years (10.56 ± 3.59)	Turkey	Turk	2.4%	1:1.4	2.6%	BCVA ≤ 20/40 in at least one eye or at least 2-lines of interocular difference
Toshihiko Matsuo *et al*.	6 to 12	Japan	Japanese	1.28%	2.5:1	0.14%	Based on the textbook classification.
Williams *et al*.	7	United Kingdom	White and Non-white	2.3%	1:3.4	3.6%	BCVA ≤ 20/40 in at least one eye or at least 2-lines of interocular difference
Josefin Ohlsson *et al*.	12 to 13	Mexico	Mexican	2.3%	1:1.8	2.5%	BCVA ≤ 20/40 or 2-line difference, presenting amblyopia factors
The Sydney Myopia Study	6	Australia	Caucasian	—	—	1.1%	BCVA of 20/40 or worse in at least one eye or at least 2-lines of interocular difference
	12			2.80%	1.3:1	0.9%	
ACES	6 to 9 (7.1 ± 0.4)	China	Center Chinese	—	—	1.0%	Unilateral: BCVA ≤ 20/32 in the worse eye or 2-line interocular difference in BCVA presenting risk factors; Bilateral: bilateral BCVA < 20/40, presenting amblyopia factors.
	12 to 13			5.0%	51:1	2.5%	
SCES	4 to 18 (9.7 ± 3.3)	China	Eastern Chinese	—	—	0.73%	BCVA ≤ 20/32 with no other reason as cause for the reduction in BCVA, presenting amblyopia factors.

YMCES = Yunnan Minority Children Eye Study; RESC = Refractive Error Study in Children; ACES = Anyang Childhood Eye Study; SCES = Shandong Children Eye Study.

Exotropia is markedly more prevalent than esotropia in Chinese school-aged children. This finding is inconsistent with previous studies in white populations, with esotropia being much more prevalent in the Whites, though the age ranges of the participants were different^5–7^. Lower hyperopia rates in Asian populations may be partially responsible^24^. Interestingly, recent studies from both Asia^13,25^ and the West^5–7^ reported similar increases in the prevalence of exotropia. The increase trend in exotropia versus esotropia ratio could be attributable to the declining occurrence of accommodative esotropia as these populations became less hyperopic. Further studies are warranted to determine the reason for this tendency with genetic and ethnic differences being taken into consideration.

With regard to amblyopia, the prevalence in our multiethnic Chinese school children was 1.43%. Compared with school-aged children in other studies (Table 5), the prevalence was higher than the rates from the Refractive Error Studies in Children (RESC) except 2.3% in Chile and 1.43% in Nepal for 2-line intraocular difference in BCVA absent in the amblyopic diagnostic criteria, which would lead to an underestimation of the prevalence by approximately half^12–19^. Following the RESC, only 86 children (0.93%) in our study in accordance with the diagnostic criteria of amblyopia, which was similar with the results from Shunyi (0.97%)^24^ and Guangzhou (0.87%)^14^.The rate of amblyopia was also higher than that found in the Shandong Children Eye Study, even though the rate 1.66% pertained only to ethnic Han children in our study^26^. When age was restricted to the same range, the rates found in our study were higher than those in other studies, including the Anyang Childhood Eye Study (1.44% versus 1.0%, 6 to 9 years)^21^ and the Sydney Myopia Study (1.52% versus 1.1% in 6 years; 1.26% versus 0.9% in 12 years)^23,27^. However, the prevalence was 1.40% lower than 2.5% in the Anyang Childhood Eye Study of children aged 12 years to 13 years^28^. The observed differences may be attributable to the variations in the definitions used in these studies. In addition, availability of vision screening programs early in life was not mentioned in some of these studies; thus, cases of amblyopia that were previously diagnosed and treated could have been discounted. In this study population, previous diagnosis and treatment of amblyopia were uncommon. Only a small number of children had undergone vision screening, with 5 of them having been previously diagnosed with amblyopia. Other children, especially those from rural regions, rarely had any form of vision screening. Therefore, this prevalence estimate reflected the natural frequency of pathology in the absence of effective surveillance and intervention, indicating the full magnitude of public health challenge. The relatively higher prevalence of amblyopia could be attributable to a lack of awareness of regular eye check-ups and the importance of using spectacles. No difference in the prevalence of amblyopia between genders was found, which was consistent with the results in previous studies. Our study found that the most common cause of amblyopia was refractive error and followed by strabismus, which was similar to the observation in the previous studies^28–30^. However, some studies identified strabismus as the main cause^23,27^.

The strengths of the study included a large and multiethnic study sample, which initially provided sufficient statistical power in detecting the potential ethnic differences of ocular diseases we are interested in. Our study also had limitations. The major limitation was that the study design was school-based rather than population-based. Although the school attendance rate in rural China has been rapidly increased in recent years, some children still could not attend schools as they are unable to afford the expenses. Thus, being unable to include these children in the study might have distorted the prevalence estimates. In addition, the diagnostic criterion for amblyopia in this study might have underestimated the prevalence, especially among children with older age.

In conclusion, no significant ethnic differences in strabismus and amblyopia among Chinese ethnic minorities were observed. Our study enriched the epidemiologic research of pediatric ophthalmology and may help ophthalmologists and optometrists to better understand the patterns of strabismus and amblyopia and potentially inform planning for school-based vision screening programs.
